# Special Issue: Next-Generation Sequencing in Tumor Diagnosis and Treatment II

**DOI:** 10.3390/diagnostics12082017

**Published:** 2022-08-20

**Authors:** Umberto Malapelle, Matteo Fassan, Dario de Biase

**Affiliations:** 1 Department of Public Health, University of Naples Federico II, 80138 Naples, Italy; 2 Surgical Pathology Unit, Department of Medicine (DIMED), University of Padua, 35122 Padua, Italy; 3 Veneto Institute of Oncology (IOV-IRCCS), 35128 Padua, Italy; 4 Department of Pharmacy and Biotechnology (FaBiT), University of Bologna, 40126 Bologna, Italy

Next-generation sequencing (NGS) allows for the sequencing of multiple genes at a very high depth of coverage. The principle of targeted therapy consists of the application of drugs targeted against well-defined molecules that play a key role in tumor progression and/or survival. Considering the continuous discovery of new molecules as a putative target or as being responsible for treatment resistance mechanisms, single-gene analyses are becoming less effective. At present, precision medicine requires multigene characterization. The introduction of NGS to molecular diagnostics has allowed us to combine the high analytical sensitivity with multigene tests [1,2,3]. The aim of this Special Issue is to focus on the application of NGS in characterizing molecular alterations in solid tumors for diagnostic, prognostic, or predictive purposes.

NGS provides a huge amount of molecular data starting from a relatively small amount of input material. However, this technique must not be considered a magician’s hat; the GIGO concept (Garbage In, Garbage Out), borrowed from computer science, is extremely valid also and above all for NGS analysis. Analyzing a total of 144 formalin-fixed and paraffin-embedded (FFPE) specimens, Chougule and colleagues performed a benchmark in quality check metrics of DNA and RNA input that should be utilized by molecular diagnostic laboratories for successful library preparation and good quality of NGS data [4]. The authors observed that samples with DIN (DNA integrity number) < 3 as well as DNA concentration < 5 ng/µL are likely to fail and that compensating the poor DNA quality with a higher DNA concentration is likely to give bad-quality NGS data. For FFPE RNA, they observed that RIN (RNA integrity number) is not an accurate quality indicator and that the RNA distribution value (DV) is a better-quality metric. Moreover, RNA library concentration is an important predictive parameter for successful RNA libraries [4]. As regards coverage and variant allele frequency cut-off for considering a “good-quality NGS sample”, the authors asserted that 250× coverage and 10% VAF (Variant Allele Frequency) have a high possibility of false-negative results and that coverage of 500× with 5% VAF is recommended for FFPE samples [4].

NGS panels would help also in characterizing the molecular alterations in clinically puzzling tumors. Malvi and colleagues have characterized a cohort of pancreatic ductal adenocarcinomas (PDAC) in order to find any molecular alterations that could be associated with histopathological features and clinical outcomes [5]. The authors have observed that the survival of patients with PDAC was related to the presence of the *TP53* and/or *KRAS* mutation. In fact, patients with PDAC harboring the concomitant KRAS/TP53 mutations had a significantly worse OS if compared to those with PDAC harboring only one of the two genes mutated or without *KRAS* and *TP53* mutations. Moreover, a dramatic prognostic difference in the *KRAS*/*TP53* double-mutated PDAC patients with an N2 stage compared to all the other patients was observed [5]. NGS can then be an alternative technique to PCR-based assays if it is not associated with extra costs, as stated by the 2020 ESMO guidelines [6], to evaluate *KRAS* and *TP53* molecular status in PDAC to improve the clinical management of the patients.

In mPDAC, the first-line therapy is based on chemotherapy (gemcitabine combined with nab-paclitaxel, or FOLFIRINOX) [7]. In 2019, the FDA (Food and Drug Administration) approved the use of PARP inhibitors (i.e., Olaparib) as maintenance for PDAC patients harboring germline *BRCA1* or *BRCA2* pathogenic mutations (gBRCAm) [8]. In this context, the assessment of *BRCA1* and *BRCA2* mutational status is nowadays crucial in PDAC patients. Bruno and colleagues evaluated the feasibility of *BRCA1/2* testing by NGS panel on a series of FFPE pancreatic tumor clinical specimens [9]. Starting from a median input DNA of 10 nanograms, they observed that 86.5% of cases were adequate for NGS analysis, with a success rate of 81.2%. Intriguingly, the failed specimens were all from tissue macrosections, characterized by a higher rate of fragmented DNA than standard sections, biopsies, and fine-needle aspirations, due to the formalin fixation procedure [9].

Liquid biopsy refers to a minimally invasive method of analysis of molecular neoplastic biomarkers performed starting from any type of patient body fluid (e.g., plasma, bile, urine, saliva, cerebrospinal fluid, and pleural effusion) [10,11]. To date, liquid biopsy analysis is routinely used in clinical practice, mainly in lung lesions, for obtaining material for molecular analyses if the “solid tissue” material is suboptimal, monitoring the treatment response, and detecting the minimal residual disease [12].

Zulato and colleagues have implemented the use of Roche’s Avenio ctDNA expanded panel in about 90 diagnostic routine non-small cell lung cancers (NSCLCs) [13]. They were able to successfully sequence 96.5% of samples. Avenio ctDNA kits demonstrated 100% sensitivity in detecting single nucleotide variants (SNVs) at VAF higher than 0.5% and high consistency in reproducibility. Moreover, they obtained matched results between tissue and liquid samples in 89% of analyzed specimens [13].

Simarro and colleagues described a case report of a patient with metastatic NSCLC who developed resistance mechanisms to the first-line EGFR-TKI treatment (dacomitinib), and to the second-line treatment with Osimertinib [14]. NGS analysis performed in liquid biopsy to characterize the resistance mechanism to second-line treatment revealed the founder deletion in exon 19 of the *EGFR* gene in concomitancy with a *TP53* deletion [14]. Further analysis allowed for the detection of a truncating mutation in the *RB1* gene, providing solid evidence of the resistance mechanism to second-line treatment with Osimertinib [14]. This study highlights the importance of implementing these high throughput molecular techniques in routine clinical practice, to understand the genomic heterogeneity of the tumor leading to personalized molecular-guided treatment.

Liquid biopsy is a useful tool not only for the management of NSCLC. Kastrisiou and colleagues had developed a targeted, cost-effective NGS gene panel that could be easily integrated in the day-to-day clinical routine starting from the plasma of patients with metastatic colorectal carcinomas (mCRC) [15]. The panel allows for the analysis of the hotspots in six clinically mCRC-relevant genes (*KRAS*, *NRAS*, *MET*, *BRAF*, *ERBB2*, and *EGFR*) with a negative and positive agreement of *RAS* Testing in Tissue and Plasma specimens of 92.8% and 81.2%, respectively [15].

As reported above, NGS multi-gene panel may be useful also to better characterize clinically interesting cases. De Falco and colleagues described two patients with CRC at different stages (pT2N0M0 and pT4cN1cM1), but both of them harbored double concurrent *KRAS* pathogenic mutations (p.G12D and p.G13D) that are normally mutually exclusive [15]. Moreover, using an NGS panel, the authors observed that the two tumors also harbored other mutations in *PIK3CA*, *SMAD4*, *NOTCH1*, *ERBB2*, and *EGFR* genes, but all of them were different in the two tumors [15].

Catino and colleagues described the clinical outcome of a patient acquiring multiple *ALK* mutations after lorlatinib treatment. In the same initial sites and at the abdominal lymph nodes and contralateral pleura, the plasma of the patient was analyzed using a multi-gene panel that confirmed an *EML4-ALK* fusion variant and also revealed the presence of *ALK* mutation p.G1202R [16]. At disease progression after ALK-TKI lorlatinib treatment, another multi-gene NGS assay was performed on cell blocks of pleural effusions revealing that the *ALK* p.G1202R mutation was still present and that another *ALK* missense mutation (p.T1151K) was found. The authors conclude that this case emphasizes the need to retest patients during the disease course and at the time of progression during ALK-TKIs therapy [16].
